# Low-Density Particleboards Modified with Blowing Agents—Characteristic and Properties

**DOI:** 10.3390/ma15134528

**Published:** 2022-06-27

**Authors:** Piotr Boruszewski, Piotr Borysiuk, Agnieszka Jankowska, Jolanta Pazik

**Affiliations:** 1 Institute of Wood Sciences and Furniture, Warsaw University of Life Sciences—SGGW, 159 Nowoursynowska St., 02-776 Warsaw, Poland; piotr_borysiuk@sggw.edu.pl (P.B.); agnieszka_jankowska@sggw.edu.pl (A.J.); 2 Fabryki Mebli “FORTE” S.A., 1 Biała St., 07-300 Ostrów Mazowiecka, Poland; jolanta.pazik@forte.com.pl

**Keywords:** raw material, blowing agent, low density, lightweight particleboard, resin content, particleboard, physical and mechanical properties

## Abstract

Although lightweight particleboards have been commercially available for years, they still have a number of disadvantages, including difficulty to process, brittleness, low impact strength, and other mechanical resistance. The aim of the paper was to determine the possibility of producing particleboards of reduced density (dedicated for furniture industry) as a result of using blowing agents from the group of hydrazides, dicarboxamides, or tetrazoles, which were modifiers of the adhesive resin used for bonding the particles of the core layer of three-layer particleboards. The concept presents the possibility of producing low-density particleboards in a standard technological process by modifying the adhesive resin, which has not been practiced by others until now. Analysis of the results of testing the particleboards properties with various types of modifiers (blowing agents), glue content (high 10%/12% and low 8%/10%), differing in glue dosing method, and different particle sizes allowed concluding that the most satisfactory effect was found in particleboards made of the variant modified with p-toluenesulfonyl hydrazide. This variant was characterised by the highest mechanical properties (bending strength, modulus elasticity, and internal bond strength) with high dimensional stability. The presented technology proposal can be applied in the industry.

## 1. Introduction

In recent years, wood-based panel industries have been undergoing rapid growth. Consequently, such an increasing demand puts strain on the wood supply, and density reduction becomes one of the topical issues in the wood-based panels industry [1]. Intensive work on reducing the density of wood-based panels has been going on since the 1990s. Most of them focus on particleboard, the importance of which is growing in the market of wood-based panels. Particleboard densities are usually in the range 600 to 750 kg/m^3^. Particleboards with densities below 600 kg/m^3^ are designated as lightweight [2] (other sources define that lightweight particleboards have a density in the range of 250–400 kg/m^3^ [3]). That makes their application in the furniture industry easier when low mass is required to facilitate transportation and assembly of the finished product (especially ready-to-assemble furniture (RTA)). So far, the production of lightweight particleboards is based on the use of low-density wood species, sandwich panels with foam cores made of polyurethane or polystyrene, or cardboard-based honeycomb cores as well as extruding tubular hollow spaces in particleboards [4].

However, lowering the board density does not always go hand in hand with lowering the costs of their production. Although it was possible to introduce many raw material substitutes for natural wood (both plant waste and new species of trees and other plants), the mechanical properties of such products are not satisfactory and differ from those of conventional products [5,6,7,8]. In addition to new wood raw materials, new binders have been introduced, the resin content has been increased, and the density profile of the product has been optimised [9,10,11,12]. The disadvantage of these solutions is often the deterioration of mechanical parameters. Such limitations are usually not present in sandwich products, for example with a honeycomb core, a foam core or made of light wood materials [13,14,15]. The factor limiting the commercialisation of new solutions is usually high production costs due to the multi-stage nature of these solutions. In recent years, however, several one-step processes have been proposed to popularise polymer foam materials, such as expanded polystyrene (EPS) or expanded microspheres (MS) [16,17,18,19,20,21].

The process of foaming the polymer contained in the composite is an effective procedure that significantly reduces the density and improves the processing of such products. In the context of particleboard, polymer foaming can refer to both foaming the binder joining wood particles and producing boards with a foam-type core [22]. In the first case, there is a reduction in the glue content in the finish product. In the second case, there is a partial replacement of both the binder and the lignocellulosic material by a polymeric foam. Thanks to this solution, products are obtained that are closer to typical boards (with high density), for example, that can be more easily joined with metal elements, such as screws. Wood-based composites with foams are also characterised by better impact strength [23] and a favourable price–quality ratio as well as strength-to-weight ratio [24]. They allow for a better product surface and sharper edges and corners. Due to the plasticising effect of the gas (as a result of its generation in the process of foaming the polymer), the production of such products takes place at a lower temperature and faster than in the case of non-foamed products, which lowers the process costs [25]. The raw material for the production of polymer foams are high-molecular polymers and various additives (fillers, auxiliaries). The forming process takes place after mixing the ingredients in the right proportions. The properties, processing, and application of the foams depend mainly on the chemical and physical properties of the polymer used. The following polymers are used in the foaming process: polystyrene (PS), polyethylene (PE), polypropylene (PP) [26,27,28], urea formaldehyde resins (UF), polyvinyl chloride (PVC), polyurethanes (PU) [29,30,31], acrylonitrile-butadiene-styrene copolymer (ABS), and phenol formaldehyde resins (PF) [27,32,33,34,35]. So far, those polymers found to be useful in wood-based plastic composites (WPC) were a success. The usage of thermoplastics for processing results from their compatibility with the wood particles favours the formation of wood plastic composite, achieving a highly efficient encapsulation of the wood particles by thermoplastic, which ensures low penetration of water into the composite, providing increasing dimensional stability [36,37].

Foamed UF resins are generally prepared by adding low-boiling alkanes such as hexane [38] or pentane [32,39] as blowing agents. As soon as their boiling point is exceeded, these blowing agents evaporate, forming cellular structures inside the resin. Due to a number of factors influencing this process, it is difficult to control the foaming effect of the resin. Hu et al. [26] used thermally expandable microspheres (TEM) to expand the UF resin. These are foaming materials in the form of capsules filled with blowing agents (low-boiling alkanes) with coatings of thermoplastic polymers such as polyacrylonitrile (PAN), poly (methyl methacrylate) (PMMA), and PU [40,41,42]. Hu et al. [26] found that the addition of TEM reduces the strength of adhesive joints. Zhao et al. [43] presented the possibilities of foaming PF resin in the production of particleboard of low density of 400 kg/m^3^), indicating that the pressure generated during the foaming of the glue ensures better contact between the wood particles and the glue, thus obtaining a better bond strength. Bi and Huang [3] showed that the influence of the blowing agent such as azodicarbonamide (AC) on the mechanical, physical, and chemical properties of particleboards of reduced density (600 kg/m^3^) is significant. Particleboards made with the use of PF resin (the degree of glue content was 12%) with 1% addition of AC to the PF resin were characterised by increased mechanical properties (in relation to bending strength, and modulus of elasticity internal bond strength), and a reduction of thickness swelling after soaking the boards in water.

Physical blowing agents do not change the composition of the system, but affect its physical condition. Such agents release gas through a physical process such as evaporation or desorption [44]. Chemical blowing agents are compounds that generate gas molecules under the influence of heat released during the production process. The decomposition of the blowing agent may be endothermic or exothermic. Exothermic releases heat during the production process, which leads to the rapid and complete breakdown of the blowing agent. A characteristic feature of exothermic blowing agents is the fact that during foaming, nitrogen is most often released, and the inert gas released by endothermic blowing agents is most often carbon dioxide. Uncommonly, only inorganic compounds are used due to the heterogeneous distribution in the polymer, often violent release of carbon dioxide, and too-high losses of the pore-forming agent [32]. In addition, the temperature range of decomposition of such compounds is wide and difficult to control, but their low price is a great advantage. In order to lower the decomposition temperature of blowing agents, it is possible to use special activators, which include, e.g., organic acids and their salts (e.g., stearic acid, zinc stearate), urea, diurea, borax, zinc oxide, hexanol, and cadmium oxide. The foaming of the polymers can take place according to two schemes. The first one involves the foaming of finished polymers, obtained in a separate stage, and relates mainly to thermoplastics. In the second method, used in the case of thermosets such as UF or epoxy resins, the foaming process takes place parallel to the polymerisation reaction [45].

Moreover, the use of foamed materials consumes quite a lot of binder. The use of in situ expandable beds improves the supersaturation of the core material compared with expanded beds. Shalbafan et al. [17] showed that the use of expandable fillers in particleboards instead of pre-expanded ones allows not only reducing the amount of binder, but also improves its effectiveness. Good mechanical parameters of the boards were obtained with a low content of the polystyrene bed (5%), but no significant improvement was observed when the content of the bed was increased to 15%, which brings economic benefits.

Luedtke and Shalbafan et al. [14] used Expancel microspheres (AkzoNobel) for the foam-molding process to lower the density of the particleboard and an expandable polystyrene deposit. In this way, the products with different structures and mechanical properties were obtained. Higher bending strength and joint strength were observed for the polystyrene foam compared with the microspheres. The favourable price also speaks as its advantage. Moreover, such boards showed an improvement in mechanical properties compared with conventional products. The possibility of a 50% reduction in the weight of the board was indicated. Shalbafan et al. [18,20] also showed a significant influence of production parameters (pressing time and temperature, foaming time) on the structure and properties of the board. 

In order to reduce the environmental effects (substitution of petroleum raw materials), Yoon et al. [46] proposed boards with polylactide foams, foamed with supercritical carbon dioxide. In addition, studies by Ganne-Chédeville and Diederichs [47] showed the benefits of replacing traditional particleboard materials with expanded polystyrene foam structures or polymethyl methacrylate or polylactide blends. The authors focused on environmental factors, including energy consumption during the production process. The use of waste polymers, especially polylactide, has proven advantageous.

The analysis of the available literature on the topic related to light particleboards indicates a fairly extensive interest in this topic by scientists, but with regard to the use of the solution presented in this paper, only Zhao et al. [43] and Bi and Huang [3] presented a convergent approach. The high level of innovativeness of the topic is confirmed by scientific research scenarios (wood sector of industry foresight). Scientific issues dealing with lightweight particleboard technology and alternative raw materials for the wood-based panels industry (including the possibility of adapting wood from fast-growing tree plantations) are currently the highest priority of science in the wood-based materials sector of European industry (P7). These issues have high potential for implementation (estimated at 4.7 points on a scale of 5.0 points) with an ever-increasing demand for results of current research [48]. The concept of producing particleboards with reduced density presented in this paper is a completely new approach to the subject. It presents the possibility of producing low-density particleboards in a standard technological process by modifying the adhesive resin (adding a blowing agent), which has not been practiced by almost anyone so far.

The objective of the presented paper was to determine the possibility of producing particleboards of reduced density (dedicated for furniture industry) as a result of using blowing agents from the group of hydrazides, dicarboxamides, or tetrazoles, which were a modifiers of the adhesive resin used for bonding the particles of the core layer of the particleboard. The selection of blowing agents used in this study resulted from the conducted preliminary research, the results of which are planned to be published in the next study.

## 2. Materials and Methods

### 2.1. Particleboards Manufacturing

As part of the experiment, three-layer particleboards with an assumed density of 520 kg/m^3^ and dimensions (width × length × thickness) of 320 × 320 × 15 mm^3^ were made. The boards were produced in 16 variants of 10 repetitions. A detailed list of assumptions for individual variants, within which three-layer particleboards were produced, is presented in the Table 1. The boards were made of industrial Scots pine (*Pinus sylvestris* L.) particles for which the average moisture contents were 4.9% (face layer) and 4.5% (core layer). The particles of the core layer within the individual variants were characterised by thickness of 0.4 mm and various length (8 mm and 4 mm), while the particles of the face layers were typical from the industrial production of particleboards. 

The particles were bonded with commercial urea formaldehyde (UF) resin (AB Achema, Jonavos, Lithuania) with a concentration of 65%, hardened with a 10% aqueous solution of ammonium sulfate (Merck KGaA, Darmstadt, Germany). The share of hardener dry weight calculated on the dry weight of the UF resin used for bonding the particles of the face and the core layer at the applied glue contents was 0.2%. Glue was dosed in two methods: pneumatic spraying and by flow dosing. In the case of boards variants from V to XVI, the UF resin was modified with the addition of blowing agents in the amount of 5% in relation to the weight of the resin at a concentration of 65%. The blowing agents used were: p-toluenesulfonyl hydrazide (group of hydrazides) (Merck KGaA, Darmstadt, Germany), azodicarbonamide (group of dicarboamides) (Merck KGaA, Darmstadt, Germany), and 5-phenyl-1H-tetrazole (group of tetrazoles) (Merck KGaA, Darmstadt, Germany). Paraffin (Polwax S.A., Jasło, Poland) in the form of an emulsion (1% of dry wood mass) was used as a hydrophobic agent. 

Mats were hand-formed from the previously bonded particles and pre-cold pressed under a pressure of 0.5 MPa for 30 s. Hot-pressing parameters were selected on the basis of industrial conditions and literature [49]. The mat pressing process was carried out using a computer-controlled laboratory press. The maximum unit pressing pressure in the adopted cycle was 2.5 MPa and was maintained until the assumed board thickness was achieved, successively reduced until the end of the assumed pressing time. The press plates temperature was 180 °C, the pressing ratio was 18 s/mm, the closing speed of the press was 2 mm/s, and the total pressing time was 270 s. Variable pressing parameters were measured automatically; the temperature of the mat core had an accuracy of ±0.01 °C, pressure had an accuracy of ±0.01 MPa, and the thickness of the mat had an accuracy of ±0.01 mm. The temperature inside the mat was measured with a Fe-CuNi thermocouple fixed in the mat core during its formation.

The research plan (Table 1) was designed based on the Taguchi method, which involves selection of response variables, identification of noise and constant factors, determination of controllable factors, selection of experimental design and data analysis method, experimental determination of optimal factors setup, and defining of expected values for dependent variables in optimised conditions. As part of the designed experiment, the boards produced within individual variants were differentiated by four factors with varying degrees of variability, i.e., modifier type—4 degrees of variation, glue content—2 degrees of variation, glue dosing—2 degrees of variation, and core particle size layer—2 levels of variation. Experimental design based on the Taguchi method provides reliable results and elimination of noise affecting final results.

### 2.2. Particleboard Properties

The property testing was preceded by particleboard surfaces calibration. In order to remove irregularities and guarantee flat parallel surfaces, the boards were sanded. The test samples were prepared in accordance with the relevant standards. Prior to testing, all samples were conditioned (before surfaces calibration) in a climate chamber at 65% (±5%) relative humidity and a temperature of 20 °C (±2 °C) until constant mass was reached. Density measurements were performed according to EN 323 [50]. The samples were square shaped, with side length of 50 mm. Density was calculated using the mass and volume of specimen after drying. Density profile studies were performed using the laboratory X-ray density analyser GreCon Da-X (Fagus-Grecon Greten GmbH & Co. KG, Alfeld-Hannover, Germany). Density measurements on the board thickness were taken with a step of 0.02 mm at a measurement speed of 0.05 mm/s. 

The bending strength (MOR) and modulus of elasticity (MOE) were determined according to the EN 310 [51] standard. Determination of internal bond strength (IB), also known as tensile strength perpendicular to the plane of the board, was performed according to EN 319 [52]. The force required to withdraw a screw (screw holding) from tested particleboards perpendicular to the surfaces (SH Ʇ) and parallel to the surface (SH II) was determined according to EN 320 [53]. The surface hardness (HB) was determined based on the recommendations of the EN 1534 [54] standard.

Thickness swelling was determined according to the method described in EN 317 [55]. The increase in thickness was evaluated after complete immersion in water for 24 h. 

The determination of each property was carried out for at least 10 replicates for a given variant of the board. The mean values of tested parameters were compared in the one-way analysis of variance (ANOVA)—Tukey’s post hoc test, in which homogeneous groups of mean values for each parameter were identified for *p* = 0.05. The significance of the influence considered variables was calculated using multi-factor ANOVA test by determining the percentage of contributing analysed factors. The experimental data were statistically analysed using STATISTICA 13.3 software (TIBCO Software Inc., Palo Alto, CA, USA).

## 3. Results and Discussion

### 3.1. Density Profile

**Figure 1:**
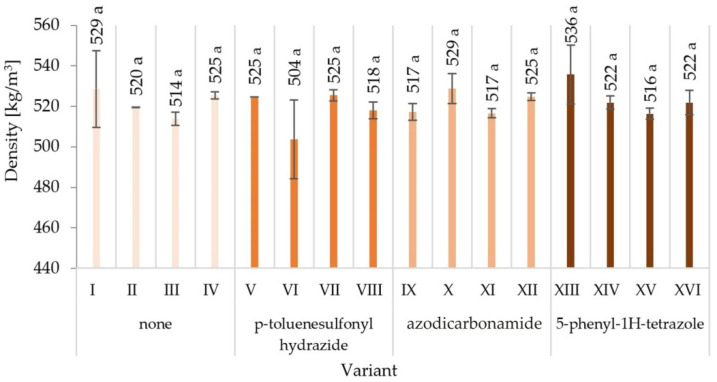
Density of particleboards produced (means and standard deviation, a, b, c, …—homogeneous groups determined by the Tukey test; different letters denote a significant difference; means followed by the same letter do not statistically differ from each other).

The highest density value for boards differing in the type of modifier used, the degree of glue content, type of glue dosing, and the size of the particles used was recorded for variant XIII—536 kg/m^3^, and the lowest for variant VI—504 kg/m^3^ (Figure 1). This range is in line with the generally accepted definition of low-density boards [2]. However, the differences in the average density of individual board variants did not exceed 10%, which allows for their comparison in terms of the examined properties. Any changes in density should not have a significant impact on the obtained relations. When analysing statistical results of the board density tests, it should be noted that they do not present different groups within individual variants—they are in one homogeneous group, which proves statistically non-significant differences between them.

**Figure 2:**
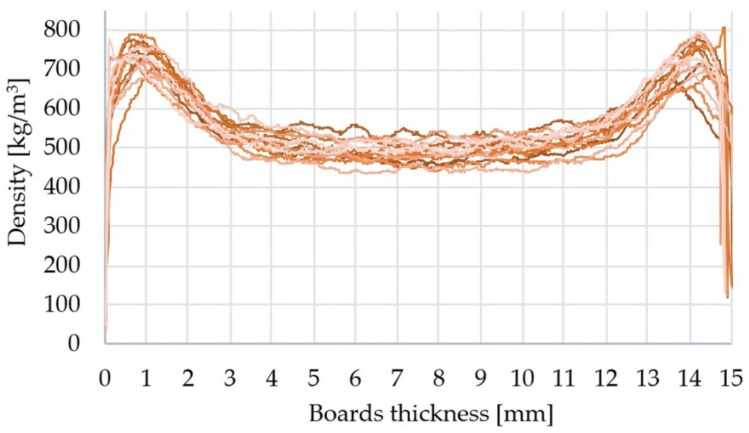
Density profiles of particleboards produced.

Figure 2 presents a summary of the density profiles on the cross-section of all variants of the manufactured boards. All of them were characterised by a similar, typical U-shaped symmetrical course. There were no significant differences between the course of the density profiles for individual board variants. U-shape profile was explained by decreasing pressure in the core as the moisture left and by a densification process in the face region within which the particles were softened by steam [56]. Wong et al. [57,58,59] and Treusch et al. [60] reported that the density profile clearly correlates with the basic properties of particleboards such as MOR, MOE, or IB. Hunt et al. [61] also showed that there is a correlation between the vertical density profile and IB test and most failures occured in the low-density core region during tests presented in this paper.

### 3.2. Mechanical Properties

**Figure 3:**
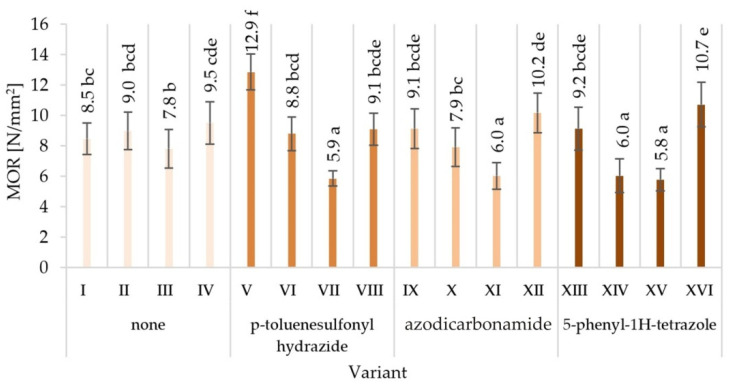
Static bending strength (MOR) of particleboards produced (means and standard deviation, a, b, c, …—homogeneous groups determined by the Tukey test; different letters denote a significant difference; means followed by the same letter do not statistically differ from each other).

**Figure 4:**
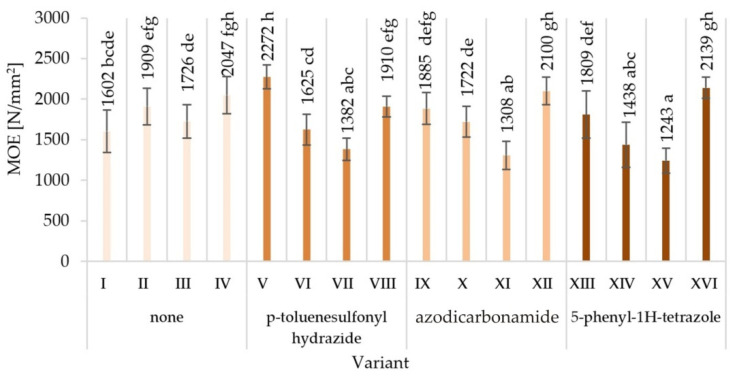
Modulus of elasticity (MOE) of particleboards produced (means and standard deviation, a, b, c, …—homogeneous groups determined by the Tukey test; different letters denote a significant difference; means followed by the same letter do not statistically differ from each other).

**Figure 5:**
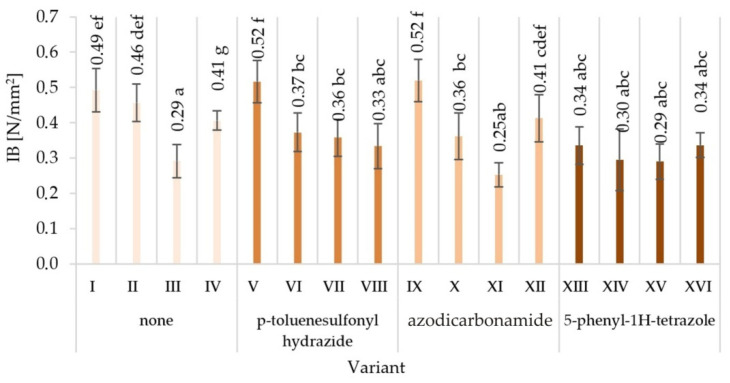
Internal bond strength (IB) of particleboards produced (means and standard deviation, a, b, c, …—homogeneous groups determined by the Tukey test; different letters denote a significant difference; means followed by the same letter do not statistically differ from each other).

**Figure 6:**
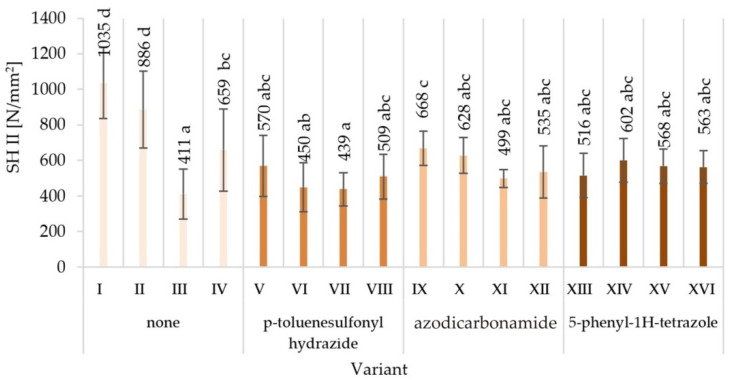
Screw holding parallel to the surface (SH II) of particleboards produced (means and standard deviation, a, b, c, …—homogeneous groups determined by the Tukey test; different letters denote a significant difference; means followed by the same letter do not statistically differ from each other).

**Figure 7:**
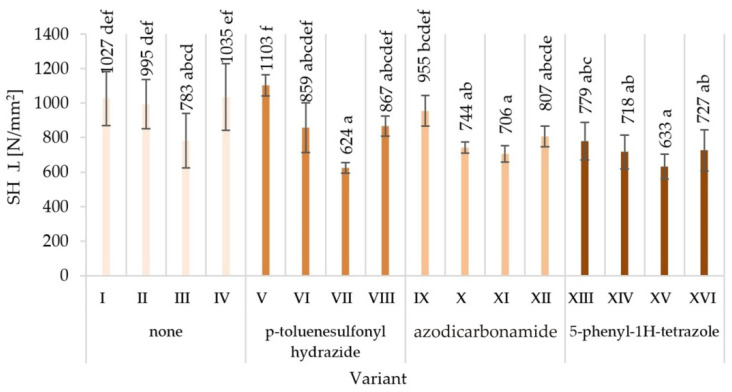
Screw holding perpendicular to the surface (SH Ʇ) of particleboards produced (means and standard deviation, a, b, c, …—homogeneous groups determined by the Tukey test; different letters denote a significant difference; means followed by the same letter do not statistically differ from each other).

**Figure 8:**
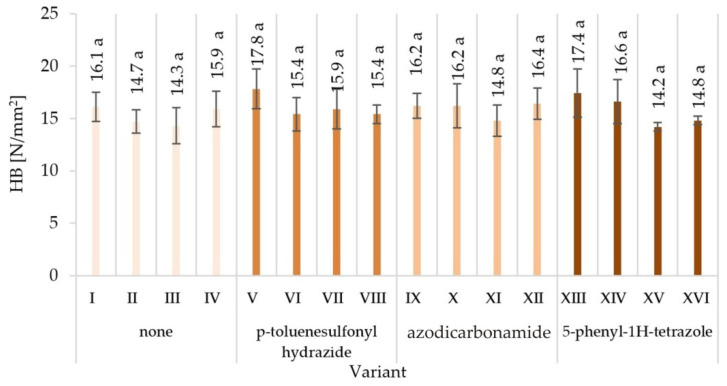
Surface hardness (HB) of particleboards produced (means and standard deviation, a, b, c, …—homogeneous groups determined by the Tukey test; different letters denote a significant difference; means followed by the same letter do not statistically differ from each other).

The obtained averaged results of determination mechanical properties of tested variants produced particleboards are presented together with the values of standard deviations in Figure 3, Figure 4, Figure 5, Figure 6, Figure 7 and Figure 8. Among the variants differing in the type of modifier used, the glue content, particle size, the type of glue dosing, and the highest value of static bending strength were characterised by variant V—12.9 N/mm^2^, followed by variant XVI—10.7 N/mm^2^. The lowest values were recorded for variant XV—5.8 N/mm^2^, VII—5.9 N/mm^2^, XI—6.0 N/mm^2^, and XIV—6.0 N/mm^2^. Similar relations were observed in the case of the value of the modulus of elasticity determined during the static bending strength test. In addition, in this case, the highest value of the modulus of elasticity in static bending was characteristic for the V variant—2272 N/mm^2^, and the lowest one for the XV variant—1243 N/mm^2^.

As a result of the comparison of the obtained averages values, it was found that the highest value of internal bond strength was shown by the variant V—0.52 N/mm^2^ and I—0.49 N/mm^2^. The lowest internal bond was characteristic for variant III and at the same time XV—0.29 N/mm^2^ and variant—0.25 N/mm^2^. Variant V was also distinguished by the highest ability to keep screws on a plane—1103 N/mm^2^. Variant IV—1035 N/mm^2^ turned out to be comparable. The least preferred variant turned out to be the variant VII—624 N/mm^2^.

The lowest surface hardness (14.2 N/mm^2^) was found in variant XV, manufactured with the use of the 5-phenyl-1H-tetrazole modifier, with glue content of 8%/10%. The highest surface hardness (17.8 N/mm^2^) was achieved in the V variant of the particleboard, manufactured with the modifier p-toluenesulfonyl hydrazide, with glue content of 10%/12%.

The observed mean values of mechanical properties in Tukey’s test were classified in different homogenous groups. Only in the case of hardness there was one homogenous group. The results of the statistical analysis provide the basis for selecting a compilation of the variables that provide the most favourable results. The highest mechanical characteristics of the V variant were not repeated in any of the other variants, indicating in the analysed comparisons that this variant stood out from the others (Figure 3, Figure 4, Figure 5, Figure 6, Figure 7 and Figure 8).

Generally, the addition of blowing agents and the increase in the degree of glue content as well as the glue dosing method contribute to the improvement of the strength parameters of the manufactured boards. These relations were only not found in the case of screw holding parallel to the surface (SH II). Among the blowing agents tested, the best results were obtained for p-toluenesulfonyl hydrazide, with a higher degree of glue content (10%/12%) and bonding particles using the pneumatic spray method (variant V). In this case, both the higher degree of glue content and the bonding of particles using the method of pneumatic spraying in combination with the use of a blowing agent resulted in better coverage of the particles surface, which translated into an increase in strength parameters. This coincides with Dunky’s indications [62], who found that the properties of the boards are influenced by the content of glue, the quality of its distribution on the surface of the particles, and the total surface of the particles covered with the glue. It is believed that the addition of glue influences the increase of the tensile strength in the direction perpendicular to the plane of the boards to a greater extent than the bending strength. The optimal amounts of glue for boards bonded with UF resins are most often indicated for core layers 4–8% and 8–14% for the face layers of the boards [63,64]. In the case of the tested variants of boards with the addition of blowing agents (variants V–XVI), both the decrease in the degree of glue content and the jet dosing of the glue to the wood particles resulted in the deterioration of the strength parameters of the boards. No analogous relations were found in the case of particleboards produced without the addition of blowing agents (variants I–IV).

Bi and Huang [3] also used azodicarbonamide for foaming adhesive resin in the production of poplar particleboards; however, they modified PF resin, which is much less popular than UF resin in the production of particleboards. The authors of ref. [3] produced particleboards of a density of 600 kg/m^3^, characterised by strength parameters that meet the requirements of EN 312 [65]. In this study, using azodicarbonamide as a modifier of adhesive resin, such satisfactory parameters of the boards were not achieved (the requirements of the EN 312 [65] standard were met by the boards from variant V, where the adhesive resin was modified with p-toluenesulfonyl hydrazide). This is most likely caused by the lower level of the assumed density of the manufactured boards as well as the fact that in the presented study the boards were made of pine particles, not poplar. Pine wood is characterised by a higher density than poplar wood, which in turn is decisive for thickening the mats during the pressing process [56]. Moreover, the additional characterisation showed that the thermal degradation of the foaming agent effectively generates the production of micro-foaming pores on the wood particles surface [3]. With regard to the tested boards, it should be stated that, in general, no effect of the change in the particle size of the internal layers on the strength parameters was observed. It is generally accepted that the size of the particles affects their mutual “fit” and the quality of their mutual bonding with glue joints. Dukarska et al. [66] showed that the particle geometry influences the bulk density of the mats as well as the degree of their compression. Medved and Resnik [67], examining the influence of the particle geometry of the face layers of boards on the bending strength, showed that the increase in particle size and the resulting decrease in the particle surface area causes a decrease in strength. Among the particles dimensions, the most important are their thickness and length [67,68]. With reference to the presented research, it is necessary to point out the multi-path influence of the particle length on the properties of the boards. On the one hand, long particles (of the same thickness) increase the bending strength as the contact surface between them increases. However, short particles contribute to an increase in the homogeneity of the board structure, and thus may increase its tensile strength [68].

When analysing the ANOVA results concerning the influence of individual tested factors on the strength properties of the boards, it should be stated that they were diversified (Table 2). The blowing agent had the strongest effect on values of IB (Figure 5), SH II (Figure 6), and SH Ʇ (Figure 7) (respectively *P* = 12.80%, 20.56%, and 26.32%). In the case of MOR (Figure 3) and MOE (Figure 4), the greatest influence was shown by glue dosing (respectively *P* = 38.62% and 40.63%) as well as type of blowing agent (respectively *P* = 4.60% and 5.88%). As previously stated, among the examined factors, the least influence was shown by particle size (*P* from 0.01% to 2.60%). This was generally a statistically insignificant impact, except for MOE. The total effect of the examined factors on the strength properties of the boards was generally (except for SH Ʇ and MOE) smaller than the influence of factors not included in this study (error above 50.00%).

### 3.3. Thickness Swelling

**Figure 9:**
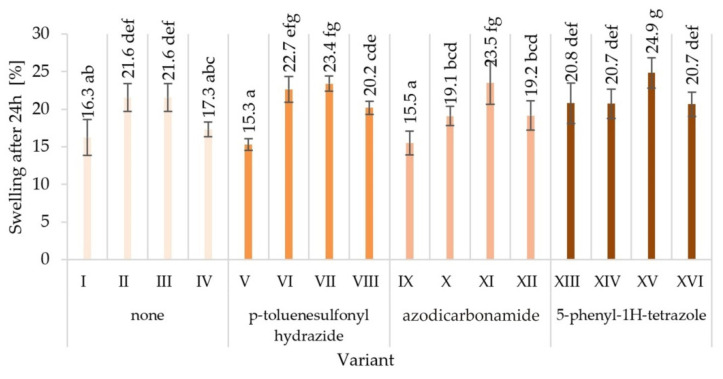
Swelling thickness changes of particleboards produced after 24 h in water (mean and standard deviation, a, b, c, …—homogeneous groups determined by the Tukey test; different letters denote a significant difference; means followed by the same letter do not statistically differ from each other (*p* < 0.05) according to Tukey’s post hoc test).

Twenty-four h soaking of the tested particleboards samples in water caused their swelling and allowed for the observation of differences in dimensional stability. The results of the swelling test for individual variants of particleboards were averaged and are presented in Figure 9. The highest dimensional stability, through the smallest range of changes, was characteristic for the V variant (swelling value was 15.3%). Variants I and IX showed an equally small scope of changes. The boards from variant XV were characterised by the lowest dimensional stability, for which the greatest swelling was recorded—24.9%. A high range of changes was also noted in the case of boards from variants II, III, VI, VII, XI, and XIII.

The thickness swelling of particleboards after soaking in water is strongly correlated with the level of their compaction [2]. Thus, it can be expected that the reduced-density particleboards will have a theoretically lower value of the swelling per thickness. However, the swelling rate of particleboards is not linearly correlated with the relative air humidity [69]. In the aspect of the analysis of particleboard swelling after soaking in water, the degree of glue content of the particles and the type of adhesive resin used for bonding the wood particles should also be taken into account. Theoretically, the higher glue content in the board, the lower swelling level [2]. On the other hand, Schulstad [70] reported that the swelling of particleboards is also related to their three-layer structure and the fact that there are free spaces in the core layer in which free water has the possibility of storage, which may in turn increase the swelling of the boards. The reduction of voids between the particles of the core layer can be achieved by using wood particles obtained from low-density species (e.g., poplar wood) for the board production [32]. Bi and Huang [3] reported that the blowing agent from the dicarboxamides group (azodicarbonamide) added to the PF resin in particleboard production technology influenced on increasing the dimensional stability of the boards and at the same time reducing the water absorption rate. It has been speculated that this phenomenon may be caused by the surface tension in the pores formed during the foaming of the resin by the blowing agent. A similar dependence was also noted in the presented study—the boards from variants V and IX had the lowest level of swelling, in which p-toluenesulfonyl hydrazide and azodicarbonamide were used to modify the UF resin, respectively, as well as, among others, higher degree of glue content of the particle.

Analysing the influence of individual tested factors on the swelling on the boards thickness after soaking in water for 24 h (Table 3), it was observed that, as in the case of strength parameters, glue dosing had the greatest impact (*P* = 38.93%). The strength of the blowing agent and glue content influence was *P* = 9.05% and 13.50%, respectively. In contrast to the strength properties, the total effect of the tested factors on the swelling on the thickness of the boards is greater than the influence of factors not included in this study (error = 35.04%). 

## 4. Conclusions

Analysis of the results of testing the particleboards properties with various types of modifiers (blowing agents), glue content (high 10%/12% and low 8%/10%), differing in glue dosing method, and different particle sizes allows for the conclusion that there was no relation between tested particleboards’ density profiles and the variables differentiating the studied variants. The highest value of static bending strength was found in particleboards made of variant V (the UF resin was modified with p-toluenesulfonyl hydrazide). Similar relations were observed for the value of the modulus of elasticity in static bending. The highest value of the tensile strength perpendicular to the planes was found in the particleboards of variant V (the UF resin was modified with p-toluenesulfonyl hydrazide).

It was observed that the hardness of particleboards depends on the glue content and the method of applying the glue on the particles. The highest hardness values were recorded for variants in which higher glue content and particles were bonded with the use of pneumatic spraying. Among the particleboards differing in the type of blowing agent used, the smallest range of dimensional changes after 24 h soaking in water was characteristic for the V-variant particleboards, in the case of which the UF resin was modified with p-toluenesulfonyl hydrazide.

Based on the result, it can be stated that for the production of particleboards with a reduced density (at the assumed level of 520 kg/m^3^), the UF resin modifier in the form of p-toluenesulfonyl hydrazide should be used, which allows for the production of particleboards with an assumed low density in laboratory conditions, meeting the basic technical requirements of the EN 312: 2011 standard (it should be noted that the standard EN 312: 2011 refers to the properties of typical particleboards with a density above 600 kg/m^3^).

## Figures and Tables

**Table 1:** Characteristics of the assumptions of individual variants within which three-layer particleboards were produced.

Variant	Type of Blowing Agent	Glue Content of the Core/Surface Layer (%)	Glue Dosing	Length of Core Layer Particles (mm)
**I**	-	10/12	pneumatic spraying	8
**II**	-	10/12	flow dosing	4
**III**	-	8/10	flow dosing	8
**IV**	-	8/10	pneumatic spraying	4
**V**	p-toluenesulfonyl hydrazide	10/12	pneumatic spraying	8
**VI**	p-toluenesulfonyl hydrazide	10/12	flow dosing	4
**VII**	p-toluenesulfonyl hydrazide	8/10	flow dosing	8
**VIII**	p-toluenesulfonyl hydrazide	8/10	pneumatic spraying	4
**IX**	azodicarbonamide	10/12	pneumatic spraying	4
**X**	azodicarbonamide	10/12	flow dosing	8
**XI**	azodicarbonamide	8/10	flow dosing	4
**XII**	azodicarbonamide	8/10	pneumatic spraying	8
**XIII**	5-phenyl-1H-tetrazole	10/12	pneumatic spraying	4
**XIV**	5-phenyl-1H-tetrazole	10/12	flow dosing	8
**XV**	5-phenyl-1H-tetrazole	8/10	flow dosing	4
**XVI**	5-phenyl-1H-tetrazole	8/10	pneumatic spraying	8

**Table 2:** ANOVA for selected factors affecting MOR, MOE, IB, SH, and HB of manufactured particleboards (*p*—probability of non-significant effects, *P*—percentage influence).

Source of Variation	MOR		MOE		IB		SH II		SH Ʇ		HB	
	*p*	*P* (%)	*p*	*P* (%)	*p*	*P* (%)	*p*	*P* (%)	*p*	*P* (%)	*p*	*P* (%)
**Blowing agent**	0.002	4.60	0.000	5.88	0.000	12.80	0.000	20.56	0.000	26.32	0.457	3.50
**Glue content**	0.000	3.75	0.012	1.80	0.002	4.78	0.000	12.38	0.000	12.80	0.008	9.86
**Particle size**	0.112	0.74	0.003	2.47	0.146	1.06	0.897	0.01	0.156	1.13	0.167	2.60
**Glue dosing**	0.000	38.62	0.000	40.63	0.000	23.26	0.009	2.98	0.000	19.75	0.014	8.54
**Error**		52.30		49.22		58.11		64.07		40.01		75.50

**Table 3:** ANOVA for selected factors affecting TS of manufactured particleboards (*p*—probability of non-significant effects, *P*—percentage influence).

Source of Variation	TS	
	*p*	*P* (%)
**Blowing agent**	0.000	9.05
**Glue content**	0.000	13.50
**Particle size**	0.001	3.48
**Glue dosing**	0.000	38.93
**Error**		35.04

## Data Availability

Not applicable.
